# 1265. Effectiveness and Durability of Dolutegravir (DTG)-Based Regimens in Older People Living with HIV (PLWH) from the Veterans Aging Cohort Study (VACS)

**DOI:** 10.1093/ofid/ofac492.1096

**Published:** 2022-12-15

**Authors:** Lei Yan, Cassidy Henegar, Kirsha Gordon, Charles Hicks, Vani Vannappagari, Amy C Justice, Mihaela Aslan

**Affiliations:** VA Clinical Epidemiology Research Center & Yale University, West Haven, Connecticut; ViiV Healthcare, Chapel Hill, North Carolina; Yale School of Medicine, New Haven, Connecticut; ViiV Healthcare, Chapel Hill, North Carolina; ViiV Healthcare, Chapel Hill, North Carolina; Yale University, West Haven, Connecticut; VA/Yale, West Haven, Connecticut

## Abstract

**Background:**

HIV management among older people living with HIV (PLWH) may be complicated by the presence of multiple comorbidities and polypharmacy. This study evaluated effectiveness and durability of modern 3-drug antiretroviral regimens among older PLWH.

**Methods:**

Using data from the Veterans Aging Cohort Study (VACS), PLWH ≥50 years old initiating a dolutegravir (DTG), bictegravir (BIC), elvitegravir (EVG), raltegravir (RAL), or darunavir (DRV)-based 3-drug regimen for the first time between January 1, 2014, and March 31, 2020 were followed from regimen initiation (baseline) until regimen discontinuation (d/c), death, loss to follow-up, or end of study (September 30, 2020). Suppression [viral load (VL)< 50 copies/mL], change in CD4 cell count, and regimen d/c were compared between regimens 6- and 12-months post-baseline using multivariable logistic or linear regression. Virologic failure (VF; 2 consecutive VLs ≥ 200 copies/ml, or 1VL ≥ 200 copies/ml followed by regimen d/c) was evaluated over 12 months. For all outcomes, DTG-based regimens were compared to each other regimen. Outcomes were stratified by treatment experience (ART-naïve and ART-experienced).

**Results:**

2,489 ART-naive (DTG: 912, BIC: 432, EVG: 751, RAL: 159, DRV: 235) and 13,810 ART-experienced (DTG: 5097, BIC: 1765, EVG: 3582, RAL: 1486, DRV: 1880) individuals were included. Included PLWH were 97% male and 30% were ≥65 years old (Table 1). For both naive and experienced PLWH, those on DTG were more likely suppressed and had greater increases in CD4 counts at 6 and 12 months compared to those on DRV or RAL (Table 2). Odds of VF did not differ by regimen for ART-naive. For ART-experienced, DTG showed reduced likelihood of VF compared to DRV and RAL. Discontinuations within the first year were higher for RAL and DRV compared to DTG. For ART-experienced PLWH, 6-month d/c was greater for DTG vs. EVG. Regardless of treatment status, no other statistical differences in outcomes were observed between DTG-, BIC-, and EVG-based regimens.

**Figure:**
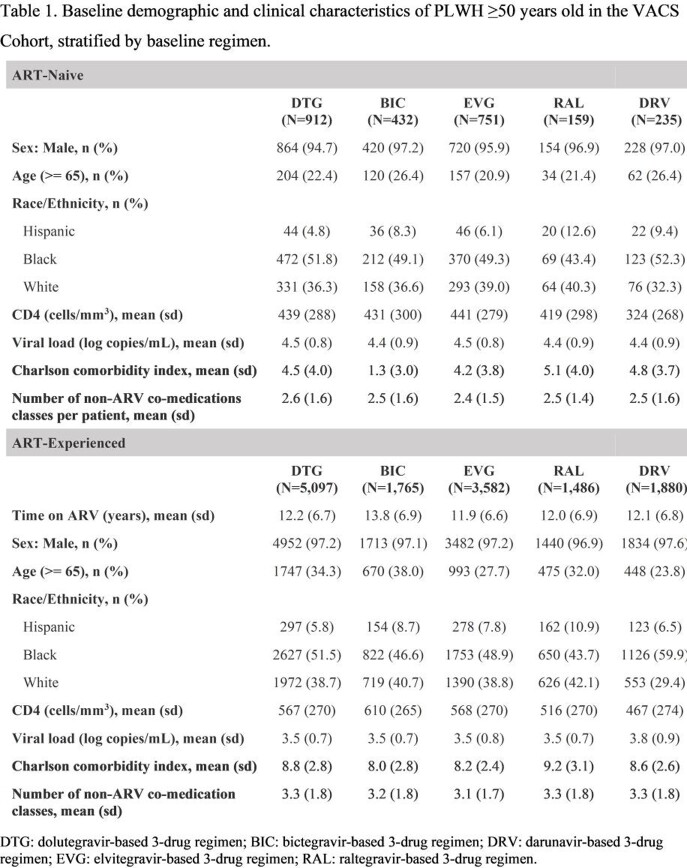

**Figure:**
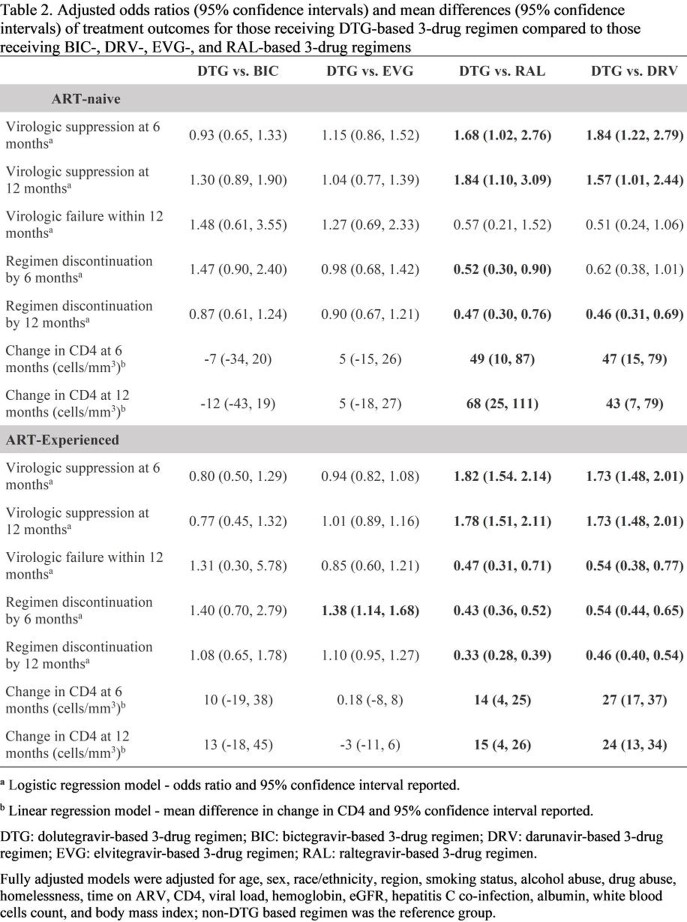

**Conclusion:**

For both ART-naïve and ART-experienced PLWH >50 years old, treatment responses during the first 12 months of follow-up were similar for those taking DTG-, BIC-, and EVG-based regimens. DTG-based regimens demonstrated greater effectiveness and durability compared to DRV- or RAL-based regimens.

**Disclosures:**

**Cassidy Henegar, PhD, MSPH**, GlaxoSmithKline: Stocks/Bonds|ViiV Healthcare: full-time employee **Charles Hicks, MD, MD**, ViiV Healthcare: I am a full time employee of ViiV Healthcare. **Vani Vannappagari, MBBS, MPH, PhD**, ViiV Healthcare: I am full time employee of ViiV Healthcare and receive GlaxoSmithKline stock as part of my compensation package|ViiV Healthcare: Stocks/Bonds.

